# SARS-CoV-2 Infection among School Population of One Developing Country. Do School Closures Protect Students and Teachers against SARS-CoV-2 Infection?

**DOI:** 10.3390/ijerph182312680

**Published:** 2021-12-01

**Authors:** Carol Bibiana Colonia, Rosanna Camerano-Ruiz, Andrés Felipe Mora-Salamanca, Ana Beatriz Vásquez-Rodríguez, Camilo Alberto Pino-Gutiérrez, Luz Amparo Pérez-Fonseca, Deidamia García-Quintero, Jennifer Ruiz-González, Iván Osejo-Villamil, Edwin Alberto Ussa-Cristiano, Fernando de la Hoz-Restrepo

**Affiliations:** 1 Epidemiología y Evaluación en Salud Pública, Departamento de Salud Pública, Facultad de Medicina, Universidad Nacional de Colombia, Bogotá 111321, Colombia; cbcolonia@unal.edu.co (C.B.C.); rcameranor@unal.edu.co (R.C.-R.); afmorasa@unal.edu.co (A.F.M.-S.); abvasquezr@unal.edu.co (A.B.V.-R.); 2 Laboratorio de Investigación en Sistemas Inteligentes, Facultad de Ingeniería, Universidad Nacional de Colombia, Bogotá 111321, Colombia; capinog@unal.edu.co; 3 Facultad de Ciencias Humanas, Universidad Nacional de Colombia, Bogotá 111321, Colombia; laperezf@unal.edu.co; 4 Secretaría de Educación Distrital, Bogotá 111321, Colombia; dgarciaq@educacionbogota.gov.co (D.G.-Q.); nruizg@educacionbogota.gov.co (J.R.-G.); iosejov@educacionbogota.gov.co (I.O.-V.); eussa@educacionbogota.gov.co (E.A.U.-C.)

**Keywords:** COVID-19, surveys and questionnaires, school teachers, students, Colombia

## Abstract

Evidence about the effectiveness of school closures as a measure to control the spread of COVID-19 is controversial. We posit that schools are not an important source of transmission; thus, we analyzed two surveillance methods: a web-based questionnaire and a telephone survey that monitored the impact of the pandemic due to COVID-19 cases in Bogotá, Colombia. We estimated the cumulative incidences for Acute Respiratory Infection (ARI) and COVID-19 for each population group. Then, we assessed the differences using the cumulative incidence ratio (CIR) and 95% confidence intervals (CI95%). The ARI incidence among students was 20.1 times higher when estimated from the telephone survey than from the online questionnaire (CIR: 20.1; CI95% 17.11–23.53). Likewise, the ARI incidence among schoolteachers was 10 times higher in the telephone survey (CIR: 9.8; CI95% 8.3–11.5). the incidence of COVID-19 among schoolteachers was 4.3 times higher than among students in the online questionnarie (CIR: 4.3, CI95%: 3.8–5.0) and 2.1 times higher in the telephone survey (CIR = 2.1, CI95%: 1.8–2.6), and this behavior was also observed in the general population data. Both methods showed a capacity to detect COVID-19 transmission among students and schoolteachers, but the telephone survey estimates were probably closer to the real incidence rate.

## 1. Introduction

The Coronavirus disease 2019 (COVID-19) pandemic has caused more than 186 million cases and over 4 million deaths worldwide since the beginning of the pandemic and up to 8 July 2021 [1]. Colombia is one of the most affected countries, reporting more than 4.4 million cases and 110,578 deaths, and Bogotá (7,743,955 inhabitants), its capital city, has reported 29.2% of the confirmed cases and 23.1% of the deaths [1,2,3,4]. On 12 March 2020, the national government declared a state of emergency throughout the country in order to prevent and control the spread of COVID-19 and mitigate its effects; the following measures were adopted: suspend events with a capacity greater than 500 people, implement hygienic measures in commercial establishments, and prohibit the transport of passengers. Specific measures for Bogotá included a total quarantine from 16 March decreed by the mayor’s office initially for two weeks and then for an indefinite period. 

There have been multiple reports studying distinct factors that affect the incidence of COVID-19 in a metropolis like Bogotá, such as the compliance of the population to the measures mandated by local and health authorities [5], the arrival of newer, more infectious strains of SARS-CoV-2 [6], particular comorbidities of individuals [7], the effectiveness of local healthcare systems [8], the socioeconomic structure of the population [9], and the effectiveness of different societal support apparatuses [10]. Nonetheless, there is scarce evidence on how the pandemic has affected school communities across the world, especially in Latin America, where most countries have closed their schools for long periods [11]. Most studies on this topic have been conducted to evaluate the impact of school transmission on the community, but none, if any, have explored what happens to a school’s population while schools are closed.

In Bogotá, the educational system had 794,598 students enrolled as of 30 September 2020 [12] and 35,415 teachers; they represented 10.7% of the total estimated population for the city. Given the volume of members in the educational system and the uncertainty about the SARS-CoV-2 transmission pattern in this population, the Secretaría de Educación Distrital (Bogotá Department of Education—SED for its initials in Spanish) has stopped all in-person activities since March 2020, and education activities have continued by online and other remote activities, like other countries have done in an effort to mitigate the impact of the SARS-CoV-2 pandemic. However, the evidence on the effectiveness of school closure to reduce COVID-19 is controversial, and the degree of benefit reported by different studies probably depends on the level of community transmission of the specific setting where the study is conducted [13]. Two regression studies have shown that this measure did have a significant effect on reducing the reproductive numbers and infection rates [14,15]. Likewise, an evaluation of the impact of nonpharmacological measures used in Hong Kong showed that the closure of schools significantly reduced the number of pediatric hospitalizations [16]. On the other hand, a model in Japan indicated that closing schools alone was not effective in mitigating the transmission of COVID-19 in the community but could be more effective if combined with other measures [17]. Additionally, another study from Israel found no evidence of increased infection between children that stayed at home compared to children attending an alternative school [18].

We posit that schools are not an important source in the transmission of COVID-19; therefore, keeping them closed does not impact community transmission. To back this hypothesis, we present analyses performed with two different methodologies implemented by the SED in order to monitor the impact of the pandemic among their population. One was an online questionnaire for self-reporting of COVID-like symptoms, and the second was a continuous random telephone survey conducted between July and 30 November 2020. We describe the main results of both strategies aiming to assess the impact of SARS-CoV-2 infection among students and schoolteachers and then compare these results with the official, publicly available COVID-19 case reports [3].

## 2. Materials and Methods

### 2.1. Study Design and Population

This study was a descriptive analysis of 2 methods—a self-administered web-based questionnaire and a population-based telephone survey—implemented to monitor the occurrence and trends of COVID-19 cases among students, schoolteachers, and administrative staff of the public school system in Bogotá (Colombia). The study population was composed of enrolled students (*n* = 794,982) and schoolteachers (*n* = 35,415) from 2191 public schools in Bogotá.

The online questionnaire was available from March 2020. Students (or their caretakers) and schoolteachers were advised to fill it out if they felt sick with COVID-19-like symptoms. The questionnaire recorded variables like name, address, ID, type of symptoms, and results of COVID tests, if any were performed. (See Supplemental Files).

The random telephone survey was conducted from 16 July to 20 November 2020. It randomly selected telephone numbers from students and teachers from a database of telephone numbers administered by the SED. The questionnaire used for the telephone survey was like the online questionnaire and recorded data on the presence of COVID-19-like symptoms and whether a COVID-19 diagnostic test was performed. A pilot study was conducted between June and July in order to assess and improve this questionnaire. The data was entered into a purpose-built web application after being checked for common mistakes affecting the data integrity. A sample size of approximately 4500 persons was estimated in order to find a 35% cumulative incidence for COVID-19 with a 95% reliability and an absolute error of 1.4%. The sample size was estimated for schoolteachers and students separately.

Additionally, we collected the official COVID-19 case registry for Bogotá from the publicly available website [3]. We selected those records that had diagnosis dates corresponding to the study period and that matched the student and schoolteacher populations by age. To calculate the incidence rates for these whole-city populations, we obtained population projections by age for Bogotá in 2020 from the national statistics department (DANE, Departamento Administrativo Nacional de Estadística) [4].

### 2.2. Statistical Analysis

We described the frequency of the symptoms, the number of COVID-19 tests reported, and the positivity rate for students and teachers. We also estimated the cumulative incidences for ARI and COVID-19. As denominators for the incidences in the groups of the self-reported questionnaire, we used the total number of students and schoolteachers registered in Bogotá schools. For the telephone survey, the incidence’s denominator was the total number of respondents in the survey. 

Finally, we compared the cumulative incidences for ARI and COVID-19 estimated for each population group between both surveillance sources and the incidence rates calculated using the official case registry. These comparisons were performed using cumulative incidence ratios (CIR) with 95% confidence intervals and a *p*-value. A two-sided Z-test was used to establish the statistical significance of the comparisons.

We used the R language and environment, Microsoft Excel and Epidata version 4.5 for the data analysis.

### 2.3. Ethical Considerations

No personal ID was available to the researchers. Participation in both information systems was voluntary. For the telephone survey, parents or caregivers provided consent for all children or adolescents selected for the survey. All of them received an explanation of the objectives of the study by the questioner and were asked to provide verbal consent to participate in the survey. Verbal consent was recorded along with the answers to the survey.

## 3. Results

### 3.1. Students

From 16 July to 20 November 2020, 5208 students completed the telephone survey. Two hundred and fifty-three (4.9%) reported respiratory symptoms; 250 COVID-19 tests were performed, and 171 (68.40%) were positive. The online questionnaire collected data from 3599 students with respiratory symptoms; 1573 tests (44%) were performed, and 1300 were positive (82.64%). Table 1 displays the number of cases by age.

A runny nose was the most-reported symptom in the telephone survey (29.47%), while fever was the most frequent in the online questionnaire (15.08%) (Table 2). 

The ARI incidence was 468.1 per 100,000 (CI95%: 438.6–453.1) from the online questionnaire and 4857.9 per 100,000 (CI95%: 4307.7–5478.3) from the telephone survey. The estimated cumulative incidence rate among students was 10.7 times higher when estimated from the telephone survey than from the online questionnaire (CIR: 10.7; CI95%: 9.44–12.18) (Table 3).

The incidence of COVID-19 was 163.7 per 100,000 (CI95%:155.0–172.8) from the online questionnaire and 3283.4 per 100,000 (CI95%: 2833.4–3804.9) from the telephone survey. The estimated cumulative incidence rate among students was 20.1 times higher when estimated from the telephone survey than from the online questionnaire (CIR: 20.1; CI95%: 17.11–23.53) (Table 3).

Using the official COVID-19 case registry [3] and the population estimates per age for Bogotá [4], the COVID-19 incidence rate for ages 0–19 was 1626.42 per 100,000 (CI95%: 1609.06–1643.96).

### 3.2. Schoolteachers

There were 5066 respondents to the telephone surveys. Six hundred and twenty-seven (12.4%) reported respiratory symptoms; 517 COVID-19 tests were performed, and 351 (67.89%) were positive. The online questionnaire collected data from 656 schoolteachers who reported respiratory symptoms; 347 COVID-19 tests were performed, and 251 (70.33%) were positive (Table 4).

In the telephone survey, the most frequent respiratory symptom reported was a runny nose (16.72%), and in the online questionnaire, it was malaise (23.66%) (Table 2).

The ARI incidence rate was 1852.3 per 100,000 (CI95%: 1717.1–1998.2) from the online questionnaire and 12,376.6 per 100,000 (CI95%: 11,502.2–13,317.5) from the telephone survey. The estimated cumulative incidence rate among students was 6.7 times higher when estimated from the telephone survey than from the online questionnaire (CIR: 6.7; CI95%: 5.9–7.5) (Table 3).

The incidence of COVID-19 was 6928.5 cases per 100,000 (CI95%: 6253.4–7664.3) from the telephone survey and 708.7 cases per 100,000 (CI95%: 626.5–801.7) from the online questionnaire. The estimated cumulative incidence was almost 10 times higher when estimated from the telephone survey than from the online questionnaire (CIR: 9.8 CI95%: 8.3–11.5) (Table 3).

Using the official COVID-19 case registry [3] and the population estimates per age for Bogotá [4], the incidence rate for ages 20–70 was 4591.51 per 100,000 (CI95%: 4573.76–4609.32).

From the online questionnaire, the cases of COVID-19 among schoolteachers were 4.3 times higher than among students (CIR: 4.3, CI95%: 3.8–5.0), while they were 2.1 higher when using data from the telephone survey (CIR: 2.1, 95% CI: 1.8–2.6). Additionally, when comparing the COVID-19 incidence rate in the official case registry [3] between the general populations aged 0–19 and 20–70, we found that the cumulative incidence was approximately 2.8 times higher for the older group than the younger group (CIR: 2.82; CI95%: 2.79–2.86).

## 4. Discussion

School closures have been a widespread measure taken all over around the globe to stop the dissemination of COVID-19/SARS-CoV-2. However, the sources of information for the efficacy of that measure are mainly ecological and modeling studies that may be inaccurate in establishing the true value of the efficacy of the measure [19,20,21]. Our study suggests that transmission in schools is only a small source of infection to the school population and that the effectiveness of closing schools to protect them seems to be small.

Our results applied to a population where most students belonged to the low- and low-middle social strata of a big city in Latin America. This means that these children tended to live in more crowded households than people in higher socioeconomic conditions, because living with extended families is more frequent in poorer neighborhoods. Additionally, poor families are more likely to live in more disadvantageous hygienic conditions at home, because public services (running water and adequate sewage disposure) are less available to them. Therefore, exposure to infectious people at home was higher in our sample than in people attending private schools.

During the study period, there were no face-to-face school activities in Bogotá; however, the impact of the SARS-CoV-2 infection was significant for the student and teacher populations. In agreement with what has been described internationally [1,22], the frequency of infection and severe disease were higher among schoolteachers and older students. We cannot attribute this difference to the social distancing behaviors of schoolteachers and students; there are several personal and environmental factors that modulate infection and social distancing behavior that we could not measure [23,24]. Notwithstanding the additional social isolation measures taken by both students and schoolteachers, the age-related risk distribution for COVID-19 infection remains; the incidence rates obtained using the official case registry [3] and the telephone survey behaved similarly with respect to age [3]. The differences in observed incidence rates between the official data and the telephone survey could be attributed to the recall bias inherent to the telephone survey, which constituted a self-report [25].

The online questionnaire was designed as a quick and routine instrument to detect cases without prior validation. This surveillance strategy is passive; thus, it has a high probability of false positives and a low sensitivity [26]. Further, the sensitivity of the telephone survey was greater to detect cases, since there was less probability of information bias, because the interviewers had prior training and used a validated tool [27]. The telephone survey presented a COVID-19 cumulative incidence rate for students 20.1 times higher than estimated from the online questionnaire. In schoolteachers, the estimated cumulative incidence rate was almost 10 times higher in the telephone survey compared to the online questionnaire. On the other hand, after the comparative analysis between the detection of COVID-19 cases in students and schoolteachers, we found that the two data sources detected more cases among schoolteachers, being, respectively, 4.3 and 2.1 times more.

The COVID-19 incidences for students and teachers estimated from the telephone survey were closer to those reported by the Bogotá Secretary of Health [3] than those from the online questionnaire. Two reasons could explain this: First, fewer COVID-19 tests than recommended have been performed to identify all possible cases of COVID-19, and telephone surveys (given their active nature) have highlighted this problem. Second, people who tested positive for COVID-19 may have been more willing to participate and complete the telephone survey than those who either had a negative result, had not even been tested, had mild symptoms, or were asymptomatic. However, the telephone survey had limitations when collecting information, such as a number of wrong phone numbers, people who rejected the phone call, and unsuccessful phone calls. We believe that telephone surveys may be a reliable source of information to monitor the health status of school populations in low-resource settings.

The most reported respiratory symptom in students and schoolteachers was a runny nose in the telephone survey; meanwhile, in the online questionnaire, it was malaise. A systematic review from Lovato et al. [28] showed that the most common respiratory symptoms in the upper airway in patients with COVID-19 were sore throat and a runny nose in a sample with a mean age of 49 years. These findings are helpful to build a sensitivity definition for suspected cases who should be encouraged to remain at home while those symptoms are present.

For schools reopening, it is essential to implement strategies at the population and school community levels to reduce the spread of COVID-19, such as: mandatory face masks, systematic testing, tracing and isolation in symptomatic individuals, and clear and continuous information for parents and schoolteachers, so that they might detect possible cases early. Schools reopening is feasible and safe following the aforementioned strategies, which could prevent and contain eventual school outbreaks [29,30,31].

## 5. Conclusions

The tendencies and dynamics of SARS-CoV-2 transmission cannot be described only from an epidemiological or clinical setting. Multiple factors must also be considered: sociocultural, biological, health services, and abiotic. The interactions of these factors determine the probability of the different outcomes of the disease (infection, morbidity, and/or mortality) at the individual, family, and group levels. For this reason, it is necessary to establish the situation or behavior of a health event of interest to the public health, such as COVID-19, an ecological analysis from the context, which allows determining the current state of the disease for the population and the differentiated measures for its control and mitigation.

Our findings suggest that the educational system should implement robust surveillance strategies during the forthcoming reopening of schools to improve the identification of active and potential cases, contain eventual school outbreaks, and support decision-making. These strategies should articulate the collection, analysis, and diffusion of information that allows the timely evaluation of the transmission dynamics at the school level while considering the diversity of the COVID-19 risk factors. We found that the lack of an adequate sampling procedure for online questionnaires represents an obstacle when comparing COVID-19 incidences between populations.

COVID-19 transmission in school settings is related to the level of community transmission; our analysis showed that, while schools remained closed during 2020, the number of cases in Bogotá increased over time, and the peaks of transmission depended more on the relaxation of restrictions and the increase in mobility in public spaces. We did not find evidence of the protective effect of school closure when comparing the COVID-19 incidence rates in students, schoolteachers, and the general population. This suggests that the closure of schools is not an effective measure to control the transmission of COVID-19.

## Figures and Tables

**Table 1:** Distribution of ARI cases in students by age group.

Age Group	Telephone Survey *n* (%)	Online Questionnaire *n* (%)
0–5	19 (7.51)	256 (7.11)
6–11	122 (48.22)	1513 (42.04)
12–18	107 (42.29)	1746 (48.56)
>19	5 (1.98)	79 (2.20)
No data	-	45 (0.14)
Total	253 (100)	3599 (100)

**Table 2:** Distribution of symptoms in the groups of interest ^1^.

Symptom	Telephone Survey		Online Questionnaire	
	Students	Schoolteachers	Students	Schoolteachers
	*n* (%)	*n* (%)	*n* (%)	*n* (%)
Runny nose or stuffy nose	28 (29.47)	148 (16.72)	1101 (11.83)	214 (10.25)
Sore throat	8 (8.42)	134 (15.14)	1349 (14.49)	332 (15.90)
Cough Fever	15 (15.79)	135 (15.25)	1273 (13.67)	325 (15.57)
	5 (5.26)	58 (6.55)	1404 (15.08)	251 (12.02)
Malaise	6 (6.32)	142 (16.05)	2386 (25.63)	494 (23.66)
New loss of taste or smell	NA	NA	483 (5.19)	164 (7.85)
Difficulty breathing or shortness of breath	26 (27.37)	114 (12.88)	576 (6.19)	184 (8.81)
Swollen hands or feet	NA	NA	48 (0.52)	28 (1.34)
Skin rash	NA	NA	60 (0.64)	10 (0.48)
Asymptomatic	NA	NA	629 (6.76)	86 (4.12)
Chest pain	4 (4.21)	65 (7.34)	NA	NA
Fatigue (tiredness)	3 (3.16)	89 (10.06)	NA	NA
Total ^2^	95 (100)	885 (100)	9309 (100)	2088 (100)

^1^ Symptoms reported in the telephone survey are based on those who had telemedicine or in-person appointments (students: *n* = 30, schoolteachers: *n* = 185). ^2^ A person may experience more than one symptom.

**Table 3:** ARI and COVID-19 incidences in students and schoolteachers.

ARI Incidence *					
Group	Telephone Survey	Online Questionnaire	CIR	CI95%	*p*-Value
Students	4857.9	453.114	10.72	9.438–12.18	<0.01
Schoolteachers	12,376.6	1570.4	6.68	5.979–7.47	<0.01
COVID-19 Incidence *					
**Group**	**Telephone Survey**	**Online Questionnaire**	**CIR**	**CI95%**	***p*** **-Value**
Students	3283.4	163.7	20.06	17.11–23.53	<0.01
Schoolteachers	6928.5	708.7	9.77	8.314–11.49	<0.01

* Incidence by 100,000.

**Table 4:** Distribution of ARI cases in schoolteachers by age group.

Age Group	Telephone Survey *n* (%)	Online Questionnaire *n* (%)
20–29	20 (3.19)	61 (9.30)
30–39	182 (29.03)	227 (34.60)
40–49	220 (35.09)	201 (30.64)
50–59	160 (25.52)	127 (19.36)
60–60	45 (7.18)	39 (5.95)
No data	-	1 (0.15)
Total	627 (100)	656 (100)

## Data Availability

Restrictions apply to the availability of these data. The data was obtained from the Secretaría de Educación Distrital, Bogotá D.C. and are available from the authors with the permission of the Secretaría de Educación Distrital, Bogotá D.C.

## Supplementary Materials

The following are available online at https://www.mdpi.com/article/10.3390/ijerph182312680/s1: File: Telephone survey questions.
