# Supplementary material for: SARS-CoV-2 Infection among School Population of One Developing Country. Do School Closures Protect Students and Teachers against SARS-CoV-2 Infection?
Source: Int J Environ Res Public Health. 2021 Dec 1;18(23):12680. doi: 10.3390/ijerph182312680 (PMC8657140; doi:10.3390/ijerph182312680)
Supplement: Supplementary file 1 [file ijerph-18-12680-s001.zip › ijerph-1450956-supplementary.pdf]

Supplemental Material. S1 (Only the data analyzed for the study are shown)

Online questionnaire “Reporte de alertas tempranas por enfermedad respiratoria”
Online questionnaire “Report of early alerts for respiratory disease”

Datos referentes a la persona sobre la que se realiza el reporte (Data referring to the person on whom the report is made)

1. Tipo de actor sobre el cual se reporta la alerta (Pregunta de selección múltiple con única respuesta)
   Type of actor about which the alert is reported (Multiple-choice question with only answer)
   - Estudiantes (Students)
   - Docentes (Schoolteachers)
   - Directivo Docente (Teaching director)
   - Docente Orientador (Guidance teacher)
   - Administrativos (Administrative)
   - Personas de servicios generales o de apoyo (General or support service staff)
   - Padre, Madre o cuidador (Father, Mother o caregiver)
   - Familiar extenso con el que se convive (Extended family member with whom you live)
2. Edad (pregunta abierta) Age (Open question)
3. Género (pregunta cerrado) Gender (closed question)
   - Femenino (Female)
   - Masculino (Male)
   - Otro (Other)
4. Localidad de residencia de la persona (pregunta abierta) Place of residence of the person (open question)

Datos de alerta (Alert data)

1. Seleccione si presenta alguno o algunos de los siguientes síntomas (Pregunta de selección múltiple con única respuesta). Select if you have any or some of the following symptoms (Multiple-choice question with only answer)
   - Secreciones nasales. Runny or stuffy nose
   - Dolor de garganta. Sore Throat
   - Tos. Cough
   - Fiebre. Fever
   - Malestar general. Malaise
   - Dificultad para respirar. Difficulty breathing or shortness of breath
   - Pérdida del sentido del gusto o el olfato. New loss of taste or smell
   - Inflamación de pies o manos. Swollen hands or feet
   - Erupciones en la piel. Skin rash
   - Asintomático. Asymptomatic
2. ¿Le han practicado prueba para COVID-19? (pregunta cerrada). Did you be tested for COVID-19? (Closed question).
   - Si (Yes)
   - No (No)

Supplemental Material. S2 (Only the data analyzed for the study are shown)

Retrospective telephone interview

Encuesta “Reporte de Alertas Tempranas por Enfermedad Respiratoria” para estudiantes, docentes y administrativos diseñada por la Universidad Nacional de Colombia

Survey "Report of Early Alerts for Respiratory Illness" for students, teachers and administrators designed by the National University of Colombia

Módulo Indicadores salud (Health indicators module)

pe_1 **Durante el último mes, ¿ha tenido síntomas de problemas respiratorios?. During the past month, have you had symptoms of breathing problems**
Si (Yes)
No (No)
Nsnr (Does not know or does not answer)

pe_1_1 **¿Consultó al médico por los síntomas de problemas respiratorios? Did you see a doctor for symptoms of breathing problems?**
Si (Yes)
No (No)
Nsnr (Do not know not answer)

pe_1_1_1**¿Por cuáles síntomas respiratorios consultó? For what respiratory symptoms did you consult?**
pe_1_1_1 Tos (Cough)
pe_1_1_1 Fiebre (Fever)
pe_1_1_1 Congestión nasal (Runny or stuffy nose)
pe_1_1_1 Dificultad respiratoria (Difficulty breathing or shortness of breath)
pe_1_1_1 Dolor torácico (Chest pain)
pe_1_1_1 Malestar general (Malaise)
pe_1_1_1 Cansancio excesivo (Fatigue-tiredness)
pe_1_1_1 Dolor de garganta (Sore Throat)
pe_1_1_1 nsnr Does not know or does not answer)

pe_5 **¿A su Hijo/a le han hecho examen médico para saber si tiene COVID-19?---¿Te han hecho algún examen médico para saber si tienes Coronavirus? Has your child had a medical exam to find out if he / she has COVID-19? --- Have you had a medical exam to find out if you have Coronavirus?**
Si (Yes)
No (No)

pe_5_1 ¿**Cuál fue el resultado? Which it was the result?**
Positivo (Positive)
Negativo (Negative)
Pendiente (Pending)
